# Sociodemographic correlates of public stigma about mental illness: a population study on Hong Kong’s Chinese population

**DOI:** 10.1186/s12888-021-03301-3

**Published:** 2021-05-29

**Authors:** Lincoln Lik Hang Lo, Yi Nam Suen, Sherry Kit Wa Chan, Min Yi Sum, Cheung Charlton, Christy Lai Ming Hui, Edwin Ho Ming Lee, Wing Chung Chang, Eric Yu Hai Chen

**Affiliations:** 1 Department of Psychiatry, University of Hong Kong, Queen Mary Hospital, Pokfulam Road, 2/F New Clinical Building, Hong Kong, SAR China; 2 State Key Laboratory of Brain and Cognitive Sciences, University of Hong Kong, Hong Kong, SAR China

**Keywords:** Public stigma, Mental health literacy, Sociodemographic correlates, Anti-stigma campaigns

## Abstract

**Background:**

Individuals with psychiatric disorders are often unwilling to seek help or often follow treatment regimens, fearing public stigma. This study identified the sociodemographic correlates of public stigma while accounting for mental health literacy and life satisfaction.

**Methods:**

This study analysed data for 1514 individuals who participated in a population-based random telephone survey conducted in 2018. Sociodemographic characteristics included gender, age, education level, and occupation. Data on public stigma, mental health literacy, and life satisfaction were also collected. Multiple linear regression was conducted to examine the effects of sociodemographic characteristics on public stigma. A moderation analysis was performed to investigate the role of age and education in the relationship between mental health literacy and public stigma.

**Results:**

Sociodemographic characteristics, such as female gender, older age, lower education, and occupation (particularly retired and homemakers), were associated with a higher public stigma. The association between public stigma and mental health literacy was the most significant among individuals aged 50 years and above with lower education levels.

**Conclusions:**

This study showed that certain population subgroups, based on their sociodemographic profile, have a higher stigma about mental illnesses. Understanding the differential effects of sociodemographic factors on public stigma is imperative to developing effective anti-stigma campaigns.

**Supplementary Information:**

The online version contains supplementary material available at 10.1186/s12888-021-03301-3.

## Background

Public stigma of mental illness prevents individuals with psychiatric disorders from seeking help and continuing with appropriate treatment [1–3]. Many studies have reported the effectiveness of anti-stigma programs, and a majority of these works have targeted occupational groups, such as students [4, 5], health care professionals [6, 7], teachers [8], and professionals in direct contact with individuals with mental illness [9–11]. However, these programs have reported short-term effects and have been proven beneficial to a limited number of people [12].

In addition to programs for specific population groups, major programs and initiatives on reducing stigma and discrimination against patients with mental illness focus on the general public [9]. While both education and contact as intervention strategies can effectively improve attitudes, research has observed differential effects for various age groups [13]. Thus, it is essential to understand the specific impact of sociodemographic characteristics on public knowledge and stigma about mental illness to guide future anti-stigma campaigns for the general public. However, few studies have conducted an in-depth analysis of the topic, and the findings have been largely inconsistent. For instance, researchers have suggested that male gender is associated with a more negative attitude towards mental illness in the United States [14], Singapore [15], and the United Kingdom [16]. Some studies have found no such gender difference [17, 18]. Other studies did not find a gender difference [19, 20]. Similarly, findings on the effects of education level on mental illness stigma remain inconsistent. Lower education levels have been associated with more negative attitudes towards mental illness in Singapore [15], the United Kingdom [16], and China [21]. In contrast, the same has been said of higher education levels for the Hong Kong Chinese population [17]. Thus, further research is needed on the impact of socioeconomic factors on mental illness stigma.

The level of mental health literacy has also been associated with public stigma [17, 21, 22]; thus, psychoeducational programs aimed at improving mental health knowledge are key intervention strategies in anti-stigma programs [9]. However, the effect of psychoeducational programs on stigma reduction appears to be small [23] and tends to vary among populations from different socioeconomic backgrounds [13]. Therefore, studies on the impact of socioeconomic factors on public stigma should account for the level of mental health literacy. In addition to basic demographics, life satisfaction, which could represent general mental wellbeing while considering life stress, is also related to mental health attitudes among the general population [18, 24]. However, studies that comprehensively explore these factors have been limited.

To address these gaps, this study primarily aims to identify the sociodemographic correlates of public stigma while accounting for mental health literacy and life satisfaction. In this study, public stigma was defined as the attitudes endorsed by the members of the general public over people with mental illnesses [25]. We have also investigated if any population subgroup may benefit more from destigmatising programs that focus on improving mental health literacy. The findings shall serve as a guide for future intervention designs.

## Methods

### Study design and procedure

This study administered a randomised telephone survey to the general population of Hong Kong from 15 January to 9 February 2018 using a computer-assisted telephone interview (CATI) system. To strengthen this study’s representativeness, the sample recruitment was based on ten gender–age strata divided according to the gender–age distribution of the general population of Hong Kong as reported in the 2016 Population By-Census [26]. A random sampling method was adopted to avoid selection bias. The approach generated two lists of telephone numbers: the first list comprised of randomly selected numbers from telephone directories, while the second list was based on the first one and was created using the plus-minus one-two method. Duplicated numbers were omitted, and all remaining numbers were randomly ordered in the final sample. Subjects were Cantonese-speaking Hong Kong residents aged 18 years and above. The analysis adopted the same procedure as that in Chan et al. [17, 27, 28].

All interviews were conducted anonymously by experienced interviewers. The calls were made on weekdays between 18:30 and 22:30 and weekends and holidays as per appointments arranged with the subjects. If more than one household member was available for the interview, the member with the most recent birthday was selected. Only respondents who answered at least 90% of the questions were considered a successful case. The whole questionnaire had 37 questions requiring 15 to 25 min for the respondent to complete. To minimise sampling bias, data collected by interviewers who failed to achieve a response rate of 40% from their assigned contact list were eliminated from the final sample. The overall response rate was calculated as the number of successful cases divided by the sum of successful cases plus effective rejection by confirmed eligible participants and incomplete cases. The expected response rate was at least 50% [29]. Every number was called five times before it was dropped as an unsuccessful contact. On-site monitoring and voice recording were used to ensure data quality.

### Data collection

Data were collected for demographic characteristics, such as gender, age, education level, and occupation. Life satisfaction was measured using a single item on an 11-point Likert scale, where 0 denotes “completely dissatisfied” and 10 is “completely satisfied” [30].

Public stigma was measured using the Chinese version of the Reported and Intended Behaviour Scale (RIBS) [5, 31]. To reduce the duration of the survey, only four items on intended behaviour towards mental illness were administered (e.g., *“In the future I am willing to live with people who have a mental illness,” “In the future, I am willing to work with people who have a mental illness”*). Each item is rated on a 5-point Likert scale, where 1 denotes “strongly agree” and 5 is “strongly disagree”. Higher RIBS scores indicate a higher level of public stigma of mental illness. The internal consistency of the four items adopted in this analysis was 0.85 in previous research [31] and 0.83 in this study.

Mental health literacy was measured using the Mental Health Knowledge Schedule (MAKS) [32]. To reduce the duration of the survey, only six items on stigma-related knowledge, such as help-seeking, recognition, support, employment, treatment, and recovery, were administered. The choices of response to each item were changed from the Likert scale to the binary scale, which reflects a true or false response. This decision was also supported by an earlier study which found that the true or false method was a more accurate method of checking knowledge about mental health than the Likert scale [32]. Higher MAKS scores indicate better mental health literacy.

### Data analysis

Statistical analyses were conducted using the Statistical Package for Social Sciences (SPSS) v.26. Descriptive statistics were computed. A multiple linear regression analysis was performed to identify significant correlates for RIBS. Only significant variables in a univariate linear regression were incorporated in the multiple linear regression models. All sociodemographic variables were tested with RIBS with MAKS and life satisfaction as covariates.

To explore if lower RIBS is associated with lower MAKS in the identified correlates, moderation analyses were performed using the PROCESS macro in SPSS (model 1) [33]. The independent and outcome variables for the analysis were mental health literacy measured by MAKS and public stigma estimated by RIBS. Several moderation models were examined using the following correlates of public stigma as the moderator: (1) dichotomous gender variable (male vs female), (2) continuous age variable, (3) three-level education variable, (4) dichotomous retirement variable (retired vs not retired), (5) dichotomous housemaker variable (housemakers vs non-housemakers) and (6) continuous life satisfaction variable.

Further moderation analysis was performed using a three-level moderator, which combined age and education. Group 1 included respondents who were younger than 50 years old and with secondary or higher education. Groups 2 and 3 included respondents aged 50 and above. Respondents in the former group completed only their primary education, while those in the latter group received secondary or higher education. As all respondents under the age of 50 years old received a minimum of secondary education, there was no grouping for younger age (i.e., < 50 years) and lower education (i.e., primary education).

## Results

Of the 2694 eligible subjects, 1514 were interviewed successfully (a response rate of 56.2%). The sample showed a similar demographic profile to that of Hong Kong’s total population in 2016 (Table 1). The mean scores for life satisfaction, MAKS, and RIBS were 7.03 (standard deviation [S.D.] = 1.81), 3.99 (S.D. = 1.32), and 9.74 (S.D. = 9.00), respectively.

**Table 1:** Demographic data of the sample

		Current study		Total population in 2016 ^a^	
		N	%	N	%
Gender	Male	719	47.5	2,846,845	47.5
	Female	795	52.5	3,152,235	52.5
Age group	18–19	35	2.3	155,498	2.6
	20–24	112	7.4	435,956	7.3
	25–29	119	7.8	462,040	7.7
	30–34	90	5.9	496,676	8.3
	35–39	146	9.6	489,828	8.2
	40–44	120	7.9	521,292	8.7
	45–49	159	10.5	538,903	9.0
	50–54	141	9.3	625,605	10.4
	55–59	116	7.7	617,468	10.3
	≥ 60	472	31.2	1,655,814	27.6
Education ^b^	Primary or below	300	19.8	1,272,280	20.6
	Secondary	716	47.4	2,858,359	46.2
	Tertiary	496	32.8	2,053,696	33.2
Occupation	Workers	575	38.0	n/a	
	Mid-level ^c^	242	16.0		
	Senior Management ^c^	89	5.9		
	Retired	279	18.4		
	Homemakers	158	10.4		
	Students	73	4.8		
	Others	98	6.5		

^a^Total population excluding foreign domestic helpers in By-Census 2016 in Hong Kong

^b^Data include participants aged 15–17, segregated data from age 18 are not available

^c^Mid-level includes office managers and professionals; Senior management includes unit heads, senior professionals, chief executive officers and directors

*SD* Standard deviation, *n/a* data not available

After controlling for life satisfaction and mental health literacy, female gender (*β* = 0.054, *p* = .03), older age groups (*β* = 0.108, *p* = .003), retirement (*β* = 0.179, *p* < .001), and homemakers (*β* = 0.093, *p* = .005) were positively associated with higher public stigma. Higher education (*β* = − 0.980, *p* < .001) was positively associated with lower public stigma. These significant factors accounted for 16.4% of the variance of the total RIBS score (Table 2).

**Table 2:** Linear regression on RIBS

	Univariate linear regression					Multiple linear regression				
	*B*	95% CI for *B*		*SE*	*β*	*B*	95% CI for *B*		*SE*	*β*
		*LCI*	*UCI*				*LCI*	*UCI*		
*Independent variables*										
Female (ref.: male)	0.565**	0.156	0.974	0.208	0.070	0.438*	0.032	0.844	0.207	0.054
Age group	0.332***	0.262	0.402	0.036	0.233	0.154**	0.052	0.256	0.052	0.108
Education	−1.367***	−1.645	−1.088	0.142	−0.240	−0.980***	−1.329	− 0.632	0.178	− 0.172
Workers ^#^	−0.650**	−1.071	−0.229	0.214	−0.078	0.413	−0.222	1.047	0.324	0.049
Mid-level ^#^	− 0.954**	−1.511	−0.397	0.284	−0.086	0.228	−0.500	0.956	0.371	0.021
Senior management ^#^	−0.700	−1.569	0.168	0.443	−0.041	*Excluded*				
Retired ^#^	2.200***	1.684	2.716	0.263	0.210	1.880***	1.120	2.640	0.387	0.179
Homemakers ^#^	1.328***	0.661	1.995	0.340	0.100	1.237**	0.373	2.101	0.440	0.093
Student ^#^	−1.847***	−2.797	−0.897	0.484	−0.098	−0.29	−1.425	0.846	0.579	−0.015
*Covariates*										
Life satisfaction	−0.157**	−0.270	−0.044	0.058	−0.070	−0.156**	−0.263	−0.048	0.055	−2.827
MAKS	−0.535***	−0.689	−0.382	0.078	−0.173	−0.824***	−0.976	−0.672	0.078	−10.634
			Constant			13.817***	12.151	15.483	0.849	
			*F* [model df, error df]			29.258 [10, 1493]				
			*p*			< .001				
			Model *R* ^2^			0.164				

*Model* “Enter” method in SPSS Statistics, *B* unstandardised regression coefficient, *CI* confidence interval, *LCI* lower CI, *UCI* upper CI, *SE* standard error of the coefficient, *β* standardised regression coefficient*, R*^2^ coefficient of determination. ^#^ The reference group was the others. * *p* < .05; **p < .01; ***p < .001

**Fig. 1:**
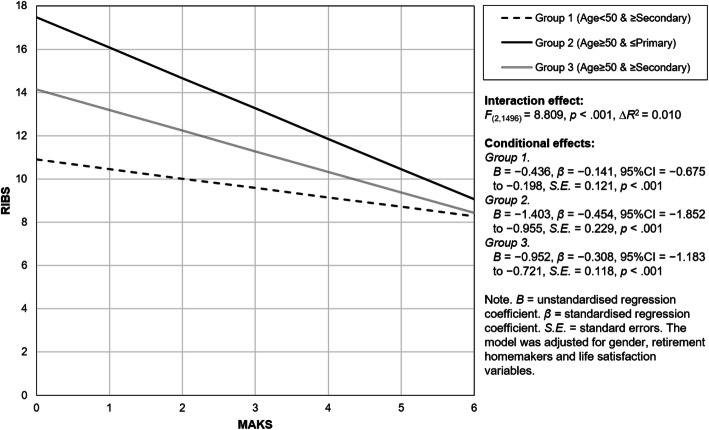
Plot of the moderation effect of age and education groups on the association between mental health literacy and public stigma

Moderation analyses found that the association between MAKS and RIBS was dependent on age and education but not on gender, retirement status, housemaker status, and life satisfaction (supplementary material 1). The differences in age and education showed significant main effects on the association (Group 2: *β* = 0.672, *p* < .001; Group 3: *β* = 0.292, *p* < .001, with the reference of group 1) (Table 3). Figure 1 depicts the significant interaction effect of age and education level on the relationship between public stigma and mental health literacy (F(2, 1496) = 8.809, ∆*R*^2^ = 0.010). Compared with those in Group 1 (younger and higher education; *β* = − 0.141, *p* < .001), the association between public stigma and mental health literacy was strongest in Group 2 (older and lower education; *β* = − 0.454, *p* < .001), followed by that in Group 3 (older and higher education; *β* = − 0.308, *p* < .001).

**Table 3:** Moderation effects of age and education on the association between mental health literacy and public stigma

Variables	*B*	95% CI for *B*		*SE*	*β*
		*LCI*	*UCI*		
MAKS	−0.436***	−0.675	−0.198	0.121	−0.141
Group 2 (ref.: group 1)	6.582***	4.196	8.968	1.216	0.672
Group 3 (ref.: group 1)	3.241***	1.879	4.604	0.695	0.292
MAKS x group 2	−9.668**	−1.474	−0.460	0.259	−0.313
MAKS x group 3	−0.516**	−0.847	−0.185	0.169	−0.167
Constant	10.827	9.509	12.145	0.672	
*F* [model df, error df]	29.067 [9, 1496]				
*p*	< .001				
Model *R* ^2^	0.149				

The model was adjusted for gender, retirement, homemakers, and life satisfaction variables. *B* unstandardised regression coefficient, *CI* confidence interval, *LCI* lower CI, *UCI* upper CI, *SE* standard error of the coefficient, *β* standardised regression coefficient*, R*^2^ coefficient of determination

**p < .01; ***p < .001

## Discussion

This study explored the sociodemographic correlates of the public stigma of mental illness using a large representative sample of the Chinese population in Hong Kong. In this sample, more knowledge about mental illness is associated with less public stigma. The findings highlighted that female gender, older age, and lower education levels are associated with significantly higher stigma about mental illness. There was no evidence of differential public stigma among populations of various occupations; however, retired individuals and homemakers are likely to have higher public stigma. The strength of the association between mental health literacy and public stigma varied by age and education level. The strongest link among people aged 50 years and above with lower education level suggested that destigmatising programs through improving mental health literacy may be more effective in this group.

In our sample, no respondents in the younger group reported a low education level. This could be because Hong Kong has nine-year free and compulsory education since 1978. It is unlikely that people born after 1970 in Hong Kong, which is about 50 years old or less, would be in low education level as it is defined as primary education (6 years) in the current study. However, it could be possible that a small proportion of people who migrated from rural China might have below secondary school education.

The finding that individuals with lower education levels are likely to show more negative stigmatising attitudes is consistent with those of previous research [34, 35]. However, this conclusion contrasts with a previous study on Hong Kong’s Chinese population [17], which suggested that higher education levels are associated with more stigmatising attitudes towards psychosis. While it is generally believed that individuals with higher education levels possess better mental health literacy [36, 37], the relationship between mental health literacy, stigma of mental illnesses and behaviour may depend on the kind of mental illness. For example, improved biological understanding of psychosis [38] and schizophrenia [39] may lead to worsening public stigma towards people with psychosis [40]. In contrast, improving knowledge about general mental illness could lead to reducing public stigma [9]. In other words, basic mental health literacy towards general mental illnesses, acquired by individuals with higher education levels, is related to a better attitude. However, the biological understanding of psychosis, which also tends to be acquired by individuals with higher education levels, worsens attitudes towards psychosis. Nonetheless, the reports were primarily from the West. Among the few Asian or local reports, improving knowledge on the nature of psychosis, but not the symptoms and treatments, may improve the public stigma of psychosis [17]. While a recent study on patient suggested that integrating biological and psychological illness attribution may improve their insight, attitude toward the healthcare professionals, and hence medication behaviour [41], such an approach may also benefit the public stigma of mental illness. After all, future investigation on the differential effect of culture on the effectiveness of the educational destigmatisation programmes should be warranted.

To the best of our knowledge, this study is the first to report a positive association between age and the public stigma of mental illness among a Chinese population. Potential generational differences in cultural beliefs may be one of the explanations of the age effect on the stigma of mental illness. This argument is supported by an earlier study highlighting the generational differences in family and gender values among the Chinese population [42]. Further research is needed to comprehensively explore the effect of variation in cultural beliefs among different generations on public stigma. This study provides further evidence that the relationship between age and public stigma of mental illness is closely related to education and mental health literacy. The moderation analysis revealed that older and less educated people have the strongest positive correlation between mental health literacy and the public stigma of mental illness. Thus, future educational destigmatising programs should target this group of individuals. These findings suggest that destigmatising interventions should adopt different approaches for people with various sociodemographics. Improving mental health literacy could be a more effective approach for older people with lower education levels.

The findings of gender effects on the public stigma of mental illness are consistent with those of prior local research, which suggested a relationship between female gender and more negative stigmatising attitudes in the Chinese population [17, 18]. However, the results contrast with those of other ethnicities, which suggested an association between male gender and more negative attitudes [14, 43, 44]. Varying cultural beliefs may explain these differences in mental health stigma. The Asian population is reportedly more susceptible to their cultural beliefs regarding mental health stigma [45]. Among the Chinese, for example, the public stigma of mental illness is often shaped by cultural meanings embedded in Confucianism, wherein the pejorative aetiological belief in mental illness is more strongly associated with the centrality of “face” [46], fear, shame, cognitive impairment, social community, consensus, and sanction [47]. Furthermore, women are at the lower end of the Confucian hierarchy. They are often expected to behave exemplarily and obey without complaint, leading to a potentially increased sense of the aforementioned negative perception of mental illness among Chinese women.

While life satisfaction was not the main focus of the correlates under examination, this study is among the few to report an association between life satisfaction and public stigma. To this effect, research has shown that life satisfaction is positively associated with all five stages in Maslow’s hierarchy of needs [48]. This association suggests that individuals with better life satisfaction are more likely to adopt a self-actualising attitude and may adopt a more positive attitude towards mental illness. However, the present findings are not in line with those of Crowe and Kim [24], who reported no evidence of an association between life satisfaction and stigma. Nevertheless, the authors selected the attitude towards mental health treatment as the outcome variable, an indicator of intended help-seeking behaviour. According to Maslow’s hierarchy of needs, such behaviours are considered ‘safety needs’, and the safety needs stage has a weaker correlation with life satisfaction than the self-actualisation stage [48].

To the best of the authors’ knowledge, this is the first population study on the sociodemographic correlates of general mental illnesses stigma in a Chinese population. However, the results should be carefully interpreted given their limitations, the first of which is the commonly reported limitations of a telephone survey [17, 18]. The most relevant issue in Hong Kong is associated with the under-coverage of the residential fixed-line telephone due to the popularity of smartphone usage [49]. Second, the findings of the current study were insufficient to delineate the public stigma toward different types of mental illness. Given that contemporary research revealed very different stigma levels depending on the particular mental illness the public is asked about [50], future investigation of public stigma toward different mental illnesses and its relationship with mental health literacy should be warranted. Third, the use of only six items on stigma-related mental health knowledge from MAKS to assess mental health literacy might have limited the cross-comparison of MAKS scores between studies. However, since this study focused on public stigma, the adopted approach was more effective for capturing stigma-related mental health knowledge. Fourth, since the survey length was limited to ensure a higher response rate, many potential confounding variables were not captured. Future studies could include sociodemographic factors, such as family structure, marital status, and income, to formulate a better model to explain the sociodemographic characteristics possibly related to the public stigma of mental illness.

### Implications

According to the World Health Organization [51], the public sees the problem of mental health as the lack of ‘vocal and powerful constituency,’ and this perception limits the extent of resources invested in the destigmatisation of mental illness. This study identified sociodemographic factors, namely, female gender, older age, especially those with lower education levels, retirement, and homemakers, related to higher stigma towards mental illness. Higher stigma in older individuals with lower education levels may be attributed to their lower mental health literacy. Thus, improving mental health knowledge can probably reduce public stigma among this population. However, the potential attributes of higher stigma in females, retirement, and housemakers remain unclear and require future investigation.

## Conclusions

The findings of this study highlighted the need to understand the differential effect of sociodemographic factors on public stigma to guide the development of effective anti-stigma campaigns. A profile-specific, anti-stigma psychoeducational program could be more cost-effective in reducing the public stigma about mental illnesses.

## Supplementary Information

**Additional file 1: Table 1.** Moderation effects of gender on the association between mental health literacy and public stigma. **Table 2.** Moderation effects of age status on the association between mental health literacy and public stigma. **Table 3.** Moderation effects of education status on the association between mental health literacy and public stigma. **Table 4.** Moderation effects of retirement status on the association between mental health literacy and public stigma. **Table 5.** Moderation effects of housemaker status on the association between mental health literacy and public stigma. **Table 6.** Moderation effects of life satisfaction on the association between mental health literacy and public stigma.

## Data Availability

The datasets used and/or analysed during the current study are available from the corresponding author on reasonable request.

## Abbreviations

B: Unstandadised regression coefficient
CATI: Computer-assisted telephone interview
RIBS: Reported and Intended Behaviour Scale
MAKS: Mental Health Knowledge Schedule
SPSS: Statistical Package for Social Sciences (SPSS)
S.D.: Standard deviation
*β*: Standardised regression coefficient
S.E.: Standard error
