# Supplementary material for: Sociodemographic correlates of public stigma about mental illness: a population study on Hong Kong’s Chinese population
Source: BMC Psychiatry. 2021 May 29;21:274. doi: 10.1186/s12888-021-03301-3 (PMC8164229; doi:10.1186/s12888-021-03301-3)
Supplement: Supplementary file 1 — Additional file 1: Table 1. Moderation effects of gender on the association between mental health literacy and public stigma. Table 2. Moderation effects of age status on the association between mental health literacy and public stigma. Table 3. Moderation effects of education status on the association between mental health literacy and public stigma. Table 4. Moderation effects of retirement status on the association between mental health literacy and public stigma. Table 5. Moderation effects of housemaker status on the association between mental health literacy and public stigma. Table 6. Moderation effects of life satisfaction on the association between mental health literacy and public stigma. [file 12888_2021_3301_MOESM1_ESM.docx]

**Supplementary material 1**

Notes for abbreviations

*B* = unstandardised regression coefficient; CI = confidence interval; *LCI* = lower CI; *UCI* = upper CI; *SE* = standard error of the coefficient; *β =* standardised regression coefficient*; df =* degree of freedom; *R*^2^ = coefficient of determination. Level of significance: * *p* < .05; ***p* < .01; ****p* < .001.

**Table 1. Moderation effects of gender on the association between mental health literacy and public stigma.**

|  |  | 95%CI for *B* | |  |  |
| --- | --- | --- | --- | --- | --- |
| Variables | *B* | *LCI* | *UCI* | *SE* | *β* |
| MAKS | −1.147*** | −1.671 | −0.622 | 0.267 | −0.371 |
| Female (ref.: male) | −0.779 | −2.112 | 0.555 | 0.680 | 0.019 |
| MAKS x gender | 0.215 | −0.102 | 0.531 | 0.161 | 0.070 |
| Constant | 12.516 | 9.894 | 15.138 | 1.337 |  |
| *F* [model df, error df] | 31.816 [8, 1332] | | | | |
| *p* | < .001 |  |  |  |  |
| Model *R* ^2^ | 0.160 |  |  |  |  |
| Note. The model was adjusted for age, education, retirement, homemakers, and life satisfaction variables. | | | | | |

**Table 2. Moderation effects of age status on the association between mental health literacy and public stigma.**

|  |  | 95%CI for *B* | |  |  |
| --- | --- | --- | --- | --- | --- |
| Variables | *B* | *LCI* | *UCI* | *SE* | *β* |
| MAKS | −0.046 | −0.512 | 0.421 | 0.238 | −0.015 |
| Age | 0.119*** | 0.082 | 0.156 | 0.019 | 0.015 |
| MAKS x age | −0.015*** | −0.023 | −0.006 | 0.004 | −0.005 |
| Constant | 8.427 | 6.047 | 10.808 | 1.214 |  |
| *F* [model df, error df] | 33.300 [8, 1332] | | | | |
| *p* | < .001 |  |  |  |  |
| Model *R* ^2^ | 0.167 |  |  |  |  |
| Note. The model was adjusted for gender, education, retirement, homemakers, and life satisfaction variables. | | | | | |

**Table 3. Moderation effects of education status on the association between mental health literacy and public stigma.**

|  |  | 95%CI for *B* | |  |  |
| --- | --- | --- | --- | --- | --- |
| Variables | *B* | *LCI* | *UCI* | *SE* | *β* |
| MAKS | −1.283*** | −1.708 | −0.858 | 0.217 | −0.415 |
| Secondary (ref.: primary) | −2.676* | −5.018 | −0.333 | 1.194 | −0.222 |
| Tertiary (ref.: primary) | −4.022*** | −6.378 | −1.666 | 1.201 | −0.324 |
| MAKS x Secondary | 0.445 | −0.043 | 0.933 | 0.249 | 0.144 |
| MAKS x Secondary | 0.679** | 0.183 | 1.176 | 0.253 | 0.220 |
| Constant | 12.984 | 10.520 | 15.448 | 1.256 |  |
| *F* [model df, error df] | 26.075 [10, 1330] | | | | |
| *p* | < .001 |  |  |  |  |
| Model *R* ^2^ | 0.164 |  |  |  |  |
| Note. The model was adjusted for gender, age, retirement, homemakers, and life satisfaction variables. | | | | | |

**Table 4. Moderation effects of retirement status on the association between mental health literacy and public stigma.**

|  |  | 95%CI for *B* | |  |  |
| --- | --- | --- | --- | --- | --- |
| Variables | *B* | *LCI* | *UCI* | *SE* | *β* |
| MAKS | −0.746*** | −0.938 | −0.553 | 0.098 | −0.241 |
| Retired (ref.: not retired) | 1.593 | −0.049 | 3.235 | 0.837 | 0.175 |
| MAKS x Retirement status | −0.222 | −0.574 | 0.131 | 0.180 | −0.072 |
| Constant | 10.994 | 9.184 | 12.804 | 0.923 |  |
| *F* [model df, error df] | 31.8779 [8, 1332] | | | | |
| *p* | < .001 |  |  |  |  |
| Model *R* ^2^ | 0.160 |  |  |  |  |
| Note. The model was adjusted for gender, age, education, homemakers, and life satisfaction variables. | | | | | |

**Table 5. Moderation effects of housemaker status on the association between mental health literacy and public stigma.**

|  |  | 95%CI for *B* | |  |  |
| --- | --- | --- | --- | --- | --- |
| Variables | *B* | *LCI* | *UCI* | *SE* | *β* |
| MAKS | −0.793*** | −0.971 | −0.616 | 0.090 | −0.257 |
| Housemaker (ref.: non-housemaker) | 1.048 | −0.880 | 2.976 | 0.983 | 0.159 |
| MAKS x Housemaker status | −0.101 | −0.536 | 0.335 | 0.222 | −0.033 |
| Constant | 11.151 | 9.355 | 12.947 | 0.916 |  |
| *F* [model df, error df] | 31.583 [8, 1332] | | | | |
| *p* | < .001 |  |  |  |  |
| Model *R* ^2^ | 0.159 |  |  |  |  |
| Note. The model was adjusted for gender, age, education, retirement, and life satisfaction variables. | | | | | |

**Table 6. Moderation effects of life satisfaction on the association between mental health literacy and public stigma.**

|  |  | 95%CI for *B* | |  |  |
| --- | --- | --- | --- | --- | --- |
| Variables | *B* | *LCI* | *UCI* | *SE* | *β* |
| MAKS | −0.414 | −0.995 | 0.168 | 0.296 | −0.134 |
| Life satisfaction | 0.154 | −0.186 | 0.494 | 0.173 | −0.018 |
| MAKS x life satisfaction | -0.056 | −0.136 | 0.023 | 0.041 | −0.018 |
| Constant | 9.706 | 6.938 | 12.474 | 1.411 |  |
| *F* [model df, error df] | 41.839 [8, 1332] | | | | |
| *p* | < .001 |  |  |  |  |
| Model *R* ^2^ | 0.161 |  |  |  |  |
| Note. The model was adjusted for gender, age, education, retirement and homemakers. | | | | | |
